# Supplementary material for: Genome-wide association study uncovers new genetic loci and candidate genes underlying seed chilling-germination in maize
Source: PeerJ. 2021 Jun 28;9:e11707. doi: 10.7717/peerj.11707 (PMC8247712; doi:10.7717/peerj.11707)
Supplement: Supplemental Information 1 [file peerj-09-11707-s001.docx]

**Supplementary Table S1.** Passport information of 300 inbred lines in this study.

| Serial number | Material number | Name | Subpopulation |
| --- | --- | --- | --- |
| 1 | SCL001 | F19 | Tropical |
| 2 | SCL002 | F06 | Tropical |
| 3 | SCL003 | Zheng58 | Tropical |
| 4 | SCL004 | Chang7-2 | Tropical |
| 5 | SCL005 | S273 | Tropical |
| 6 | SCL006 | Su1611 | Tropical |
| 7 | SCL007 | YA8201 | Tropical |
| 8 | SCL008 | 5311 | Tropical |
| 9 | SCL010 | 5Gong | Tropical |
| 10 | SCL011 | 21A | Tropical |
| 11 | SCL012 | SN8-1-1 | Tropical |
| 12 | SCL013 | Jiao51 | Tropical |
| 13 | SCL014 | V37 | Tropical |
| 14 | SCL015 | Mian7317 | NSS |
| 15 | SCL016 | Mian723 | SS |
| 16 | SCL017 | 7141_5 | Tropical |
| 17 | SCL018 | NZ013-1 | SS |
| 18 | SCL019 | Yu561 | Tropical |
| 19 | SCL020 | H08-155 | Tropical |
| 20 | SCL021 | K169R | SS |
| 21 | SCL023 | M14 | Tropical |
| 22 | SCL024 | Y8G | SS |
| 23 | SCL025 | B151 | SS |
| 24 | SCL026 | 618 | SS |
| 25 | SCL027 | 141 | SS |
| 26 | SCL028 | ShuangM9 | Tropical |
| 27 | SCL029 | P953 | SS |
| 28 | SCL030 | LN8 | NSS |
| 29 | SCL032 | En1824 | NSS |
| 30 | SCL033 | ZYDH381-1 | SS |
| 31 | SCL034 | Su95-1 | NSS |
| 32 | SCL035 | De2010-1 | SS |
| 33 | SCL036 | End28 | SS |
| 34 | SCL038 | Mian715 | SS |
| 35 | SCL039 | BS08-565 | SS |
| 36 | SCL041 | 21209 | Tropical |
| 37 | SCL042 | De12 | SS |
| 38 | SCL043 | TD1 | Tropical |
| 39 | SCL045 | Jing07-4 | Tropical |
| 40 | SCL046 | M89 | NSS |
| 41 | SCL047 | H127RE | SS |
| 42 | SCL048 | MH9 | Tropical |
| 43 | SCL049 | NZ925 | Tropical |
| 44 | SCL050 | BS1074 | Tropical |
| 45 | SCL051 | Lin-1 | Tropical |
| 46 | SCL052 | Yu9537 | Tropical |
| 47 | SCL053 | JF52-3 | Tropical |
| 48 | SCL054 | S7913 | Tropical |
| 49 | SCL055 | DH29 | SS |
| 50 | SCL056 | Mo17 | BSSS |
| 51 | SCL059 | 2142 | SS |
| 52 | SCL060 | 205-11 | Tropical |
| 53 | SCL061 | ZJ-3 | Tropical |
| 54 | SCL063 | LSC117 | Tropical |
| 55 | SCL064 | LSC107 | SS |
| 56 | SCL066 | Nan09530 | SS |
| 57 | SCL067 | M232 | Tropical |
| 58 | SCL068 | Du321 | NSS |
| 59 | SCL069 | Chuan273 | SS |
| 60 | SCL070 | Nan381 | Tropical |
| 61 | SCL071 | 7854 | NSS |
| 62 | SCL072 | 18-9-101 | Tropical |
| 63 | SCL074 | Q78 | SS |
| 64 | SCL075 | Nan637 | Tropical |
| 65 | SCL076 | Nan202 | SS |
| 66 | SCL077 | 78599-211 | SS |
| 67 | SCL079 | JS0251 | Tropical |
| 68 | SCL080 | W284 | Tropical |
| 69 | SCL081 | Zheng28 | NSS |
| 70 | SCL082 | Zheng22 | NSS |
| 71 | SCL084 | Dan3130 | SS |
| 72 | SCL085 | LC955 | Tropical |
| 73 | SCL087 | LJ-2 | Tropical |
| 74 | SCL088 | Liao68 | SS |
| 75 | SCL089 | Liao7996 | NSS |
| 76 | SCL090 | Liao3053 | SS |
| 77 | SCL091 | Liao5144 | NSS |
| 78 | SCL092 | Liao7890 | NSS |
| 79 | SCL094 | Wa138 | Tropical |
| 80 | SCL095 | Ji1037 | Tropical |
| 81 | SCL097 | ZM28 | SS |
| 82 | SCL098 | 891 | SS |
| 83 | SCL099 | K22 | NSS |
| 84 | SCL100 | Dan9046 | NSS |
| 85 | SCL102 | CN9802 | NSS |
| 86 | SCL103 | Dan598 | Tropical |
| 87 | SCL104 | Shen135 | SS |
| 88 | SCL105 | Shen136 | SS |
| 89 | SCL106 | Shen137 | SS |
| 90 | SCL107 | SH15 | SS |
| 91 | SCL108 | 871 | SS |
| 92 | SCL109 | P138 | SS |
| 93 | SCL110 | Qi319 | SS |
| 94 | SCL111 | 178 | SS |
| 95 | SCL112 | 7327 | Tropical |
| 96 | SCL113 | YS0 | SS |
| 97 | SCL114 | Ye478 | NSS |
| 98 | SCL115 | 698-3 | SS |
| 99 | SCL116 | Huangjin59 | SS |
| 100 | SCL117 | JH59 | SS |
| 101 | SCL118 | Dan340 | Tropical |
| 102 | SCL120 | CA211 | Tropical |
| 103 | SCL121 | DYS | NSS |
| 104 | SCL122 | Liao6082 | NSS |
| 105 | SCL123 | Lu2458 | NSS |
| 106 | SCL124 | Ji477 | NSS |
| 107 | SCL125 | Zheng29 | NSS |
| 108 | SCL126 | ZH64 | Tropical |
| 109 | SCL127 | H921 | SS |
| 110 | SCL128 | 1572 | NSS |
| 111 | SCL129 | Ji992 | Tropical |
| 112 | SCL130 | 9614 | NSS |
| 113 | SCL131 | 434 | Tropical |
| 114 | SCL132 | H10 | Tropical |
| 115 | SCL133 | 4379 | Tropical |
| 116 | SCL134 | 4011 | Tropical |
| 117 | SCL135 | HuangC | NSS |
| 118 | SCL137 | 9HT1804 | Tropical |
| 119 | SCL138 | M165 | NSS |
| 120 | SCL139 | H21 | Tropical |
| 121 | SCL140 | 4866 | NSS |
| 122 | SCL141 | 07G83 | Tropical |
| 123 | SCL142 | LJS-1 | SS |
| 124 | SCL143 | Yi99-19 | Tropical |
| 125 | SCL144 | LZM004 | SS |
| 126 | SCL145 | LZM05-1-1 | Tropical |
| 127 | SCL146 | LZM6-1-1 | Tropical |
| 128 | SCL147 | LZM025 | SS |
| 129 | SCL148 | W8071 | Tropical |
| 130 | SCL149 | M11 | Tropical |
| 131 | SCL150 | 1221CL8 | Tropical |
| 132 | SCL151 | QBII-1 | Tropical |
| 133 | SCL152 | 1221CL11 | Tropical |
| 134 | SCL153 | ML1108 | NSS |
| 135 | SCL154 | ML1120 | SS |
| 136 | SCL155 | W7475 | SS |
| 137 | SCL156 | Liao147-8 | SS |
| 138 | SCL157 | PN0504-8 | NSS |
| 139 | SCL158 | KS001 | NSS |
| 140 | SCL160 | 1212638 | NSS |
| 141 | SCL162 | SAM3001 | SS |
| 142 | SCL163 | 975-12 | SS |
| 143 | SCL164 | 18-599 | SS |
| 144 | SCL165 | SCML202 | SS |
| 145 | SCL166 | SCML1950 | NSS |
| 146 | SCL167 | 08-641 | Tropical |
| 147 | SCL168 | 17564 | Tropical |
| 148 | SCL169 | SCML103 | SS |
| 149 | SCL171 | SCML203 | SS |
| 150 | SCL172 | LH8012 | Tropical |
| 151 | SCL173 | 9LB050 | Tropical |
| 152 | SCL174 | TM012 | SS |
| 153 | SCL176 | 975-13 | SS |
| 154 | SCL177 | YZ15BC-2 | Tropical |
| 155 | SCL178 | SCML2054 | NSS |
| 156 | SCL179 | 10WRB115 | SS |
| 157 | SCL180 | 10WRA120 | NSS |
| 158 | SCL181 | 08WSC51 | NSS |
| 159 | SCL182 | 08WSC179 | SS |
| 160 | SCL183 | 08WSC187 | SS |
| 161 | SCL184 | 08WSC200 | SS |
| 162 | SCL185 | 08WSC204 | SS |
| 163 | SCL186 | 08WSC237 | SS |
| 164 | SCL187 | 08WSC257 | SS |
| 165 | SCL189 | CIMMYT-1 | Tropical |
| 166 | SCL190 | GP30-1 | Tropical |
| 167 | SCL191 | GP66-1 | SS |
| 168 | SCL192 | S37 | Tropical |
| 169 | SCL193 | T32 | Tropical |
| 170 | SCL195 | BJ005 | NSS |
| 171 | SCL196 | 66781 | SS |
| 172 | SCL197 | GD003 | Tropical |
| 173 | SCL198 | 03FLUSA10 | Tropical |
| 174 | SCL199 | XS021 | Tropical |
| 175 | SCL200 | 9HT1736 | Tropical |
| 176 | SCL201 | 81565 | Tropical |
| 177 | SCL202 | ZD0502-23111 | Tropical |
| 178 | SCL203 | 09YT20919 | Tropical |
| 179 | SCL204 | LH7556 | SS |
| 180 | SCL205 | Mian04185-4 | Tropical |
| 181 | SCL206 | Mian04185-8 | Tropical |
| 182 | SCL208 | SCML2031 | SS |
| 183 | SCL209 | TY30331-3 | Tropical |
| 184 | SCL210 | TY30331-2 | Tropical |
| 185 | SCL211 | QA | NSS |
| 186 | SCL212 | KS003 | Tropical |
| 187 | SCL213 | HL5049 | Tropical |
| 188 | SCL214 | HL5054 | SS |
| 189 | SCL215 | LLF-08 | Tropical |
| 190 | SCL216 | ZD808-1 | SS |
| 191 | SCL217 | 510317 | SS |
| 192 | SCL218 | Dan4245 | NSS |
| 193 | SCL219 | PZ1010-2 | SS |
| 194 | SCL220 | CMY093288 | Tropical |
| 195 | SCL221 | JD7275 | Tropical |
| 196 | SCL222 | JY01-3 | SS |
| 197 | SCL223 | Y0827 | NSS |
| 198 | SCL225 | Y1015 | Tropical |
| 199 | SCL226 | Y1018 | Tropical |
| 200 | SCL227 | Y1021 | NSS |
| 201 | SCL228 | Y1022 | SS |
| 202 | SCL229 | Y1032W | SS |
| 203 | SCL230 | Y1032R | SS |
| 204 | SCL231 | Y1035 | SS |
| 205 | SCL233 | Y1111 | SS |
| 206 | SCL234 | Y1127 | Tropical |
| 207 | SCL236 | L31 | SS |
| 208 | SCL238 | Y1005 | Tropical |
| 209 | SCL239 | Y0826 | Tropical |
| 210 | SCL240 | 77 | Tropical |
| 211 | SCL241 | BML1256 | Tropical |
| 212 | SCL242 | P801 | Tropical |
| 213 | SCL243 | 10WRC64 | SS |
| 214 | SCL244 | LX312 | Tropical |
| 215 | SCL245 | PI43W | Tropical |
| 216 | SCL246 | W8199 | SS |
| 217 | SCL247 | Y1114 | Tropical |
| 218 | SCL249 | SAM31152A | NSS |
| 219 | SCL250 | DH3732 | SS |
| 220 | SCL251 | BML1275 | Tropical |
| 221 | SCL252 | XBY13563 | Tropical |
| 222 | SCL253 | BML1269 | SS |
| 223 | SCL254 | Y1216 | SS |
| 224 | SCL255 | LM-6 | Tropical |
| 225 | SCL256 | 10GY6057 | SS |
| 226 | SCL257 | BML1243 | NSS |
| 227 | SCL258 | BML1234 | Tropical |
| 228 | SCL259 | Y1224 | Tropical |
| 229 | SCL260 | 1217 8107 | SS |
| 230 | SCL261 | BML1228 | Tropical |
| 231 | SCL262 | 08WSC166 | Tropical |
| 232 | SCL263 | LX350 | Tropical |
| 233 | SCL264 | Y1217 | SS |
| 234 | SCL265 | XBY2193 | Tropical |
| 235 | SCL266 | 10GY92-121 | Tropical |
| 236 | SCL267 | 10GY76-111 | Tropical |
| 237 | SCL268 | U8112 | NSS |
| 238 | SCL270 | C2010-3 | SS |
| 239 | SCL272 | LS-22 | Tropical |
| 240 | SCL273 | CD30M | SS |
| 241 | SCL274 | LZM009 | SS |
| 242 | SCL275 | XH05 | SS |
| 243 | SCL277 | 06WAM110 | Tropical |
| 244 | SCL281 | GCML152 | Tropical |
| 245 | SCL282 | GCML157 | Tropical |
| 246 | SCL287 | 65232B | NSS |
| 247 | SCL288 | CLRCW48 | Tropical |
| 248 | SCL289 | CLWN201 | Tropical |
| 249 | SCL291 | CLWN226 | Tropical |
| 250 | SCL292 | CLWN227 | Tropical |
| 251 | SCL293 | CLWN247 | Tropical |
| 252 | SCL294 | CLWN250 | Tropical |
| 253 | SCL295 | CLWN251 | Tropical |
| 254 | SCL298 | CL02720 | Tropical |
| 255 | SCL300 | CLRCY041 | Tropical |
| 256 | SCL301 | CLYN214 | Tropical |
| 257 | SCL303 | CML268 | Tropical |
| 258 | SCL304 | CML282 | Tropical |
| 259 | SCL306 | CML308 | Tropical |
| 260 | SCL307 | CML379 | Tropical |
| 261 | SCL310 | TL98A1709-20 | Tropical |
| 262 | SCL311 | TL96B | Tropical |
| 263 | SCL312 | 98WV9 | Tropical |
| 264 | SCL316 | SW01D1058-2 | Tropical |
| 265 | SCL317 | SW01D1058-7 | Tropical |
| 266 | SCL318 | BANTAN2003 | Tropical |
| 267 | SCL319 | CG698C102 | SS |
| 268 | SCL320 | CG921 | SS |
| 269 | SCL324 | K305 | Tropical |
| 270 | SCL325 | 811 | SS |
| 271 | SCL326 | K363 | NSS |
| 272 | SCL329 | B0069 | SS |
| 273 | SCL330 | 81565 | Tropical |
| 274 | SCL331 | LYC-1 | Tropical |
| 275 | SCL332 | WZ-1 | SS |
| 276 | SCL333 | PB80 | NSS |
| 277 | SCL335 | 793 | NSS |
| 278 | SCL336 | W8034 | NSS |
| 279 | SCL337 | PHV63 | NSS |
| 280 | SCL339 | 2369 | NSS |
| 281 | SCL340 | 87916W | NSS |
| 282 | SCL341 | PHW52 | NSS |
| 283 | SCL342 | CA1108 | Tropical |
| 284 | SCL343 | Nan21-3 | Tropical |
| 285 | SCL345 | PH4CV | Tropical |
| 286 | SCL346 | Xun9058 | NSS |
| 287 | SCL347 | LX9801 | Tropical |
| 288 | SCL348 | C24 | SS |
| 289 | SCL349 | C09-1 | Tropical |
| 290 | SCL350 | L2010-3 | Tropical |
| 291 | SCL351 | ZNC-4 | Tropical |
| 292 | SCL352 | Qi533 | SS |
| 293 | SCL353 | L6201 | Tropical |
| 294 | SCL354 | LSC127 | Tropical |
| 295 | SCL355 | W30 | SS |
| 296 | SCL356 | B73 | NSS |
| 297 | SCL357 | B047 | SS |
| 298 | SCL358 | W3189 | SS |
| 299 | SCL359 | 9782 | Tropical |
| 300 | SCL360 | 646 | Tropical |

SS, Stiff Stalk;

NSS, Non-Stiff Stalk
