# Supplementary material for: Genome-wide association study uncovers new genetic loci and candidate genes underlying seed chilling-germination in maize
Source: PeerJ. 2021 Jun 28;9:e11707. doi: 10.7717/peerj.11707 (PMC8247712; doi:10.7717/peerj.11707)
Supplement: Supplemental Information 2 [file peerj-09-11707-s002.docx]

**Supplementary Table S2.** Primers used for PCR amplification of candidate genes.

| Primer | Forward/Reverse | Sequence of primers |
| --- | --- | --- |
| *Zm00001d050021* | Forward | GTGCAAGATAAACGGCAAGC |
| *Zm00001d050021* | Reverse | CACATGCCATGAAGGAACAC |
| *Zm00001d010454* | Forward | GGGCGTGGTTGATAAGACAT |
| *Zm00001d010454* | Reverse | AACCCCCAAATCCTCAAATC |
| *Zm00001d010458* | Forward | CCAGGGAATGTTTCCAACAC |
| *Zm00001d010458* | Reverse | CGGGCAACATAGTTCCAGAT |
| *Zm00001d010459* | Forward | TTTTTCTCCATACCACCCGTA |
| *Zm00001d010459* | Reverse | TCTACAGGAGACATCCGAAGC |
