# Supplementary material for: Genome-wide association study uncovers new genetic loci and candidate genes underlying seed chilling-germination in maize
Source: PeerJ. 2021 Jun 28;9:e11707. doi: 10.7717/peerj.11707 (PMC8247712; doi:10.7717/peerj.11707)
Supplement: Supplemental Information 3 [file peerj-09-11707-s003.docx]

**Supplementary Table S3.** Primers used for qRT-PCR amplification of candidate genes.

| Gene name | Forward primer (5'-3') | Reverse primer (5'-3') |
| --- | --- | --- |
| *Zm00001d050021* | AGCCTACGAGGAGAACGATG | GCCTCAGGCTCTCCAAGATA |
| *Zm00001d010454* | TACATCAACCCCTCCTCTGG | CCTGCTGAACCCACACTTTT |
| *Zm00001d010458* | TTGGTTCAAATGGGGTCAAT | GAACAAGGCCTCCATCATGT |
| *Zm00001d010459* | AGAATGCCACCACCACAAAT | GTTTGTGGCAACCAAGGACT |
| *ZmActin1* | TTGTCCTCAGTGGGGGATCT | CTTTCAGGTGGCGCAATCAC |
