# Supplementary material for: Genome-wide association study uncovers new genetic loci and candidate genes underlying seed chilling-germination in maize
Source: PeerJ. 2021 Jun 28;9:e11707. doi: 10.7717/peerj.11707 (PMC8247712; doi:10.7717/peerj.11707)
Supplement: Supplemental Information 4 [file peerj-09-11707-s004.docx]

**Supplementary Table S4.** List of gene models within LD regions of the significant SNPs identified by GWAS.

| Candidate gene | Chr. | Interval (bp) | Functional annotations |
| --- | --- | --- | --- |
| *Zm00001d032350* | 1 | 223,440,445-223,444,783 | Unknown |
| *Zm00001d032356* | 1 | 223,602,477-223,611,036 | Wall-associated receptor kinase 3 |
| *Zm00001d032358* | 1 | 223,748,469-223,751,668 | Auxin-responsive family protein |
| *Zm00001d032357* | 1 | 223,732,522-223,733,692 | Protein aq_1857 |
| *Zm00001d032353* | 1 | 223,545,282-223,577,431 | Blue-light receptor phototropin 2 |
| *Zm00001d005182* | 2 | 162,854,863-162,858,098 | Charged multivesicular body protein 2a |
| *Zm00001d005196* | 2 | 163,133,617-163,134,078 | Unknown |
| *Zm00001d005190* | 2 | 163,038,225-163,041,426 | Cadmium/zinc-transporting atpase hma2 |
| *Zm00001d005193* | 2 | 163,079,620-163,082,144 | Protein lurp1 |
| *Zm00001d005188* | 2 | 163,003,511-163,010,881 | Cytochrome b5 domain-containing protein rlf |
| *Zm00001d005185* | 2 | 162,911,443-162,914,557 | Phd finger protein alfin-like 2 |
| *Zm00001d005189* | 2 | 163,016,710-163,020,248 | Cadmium/zinc-transporting atpase hma2 |
| *Zm00001d005184* | 2 | 162,902,441-162,909,949 | Unknown |
| *Zm00001d032111* | 2 | 213,313,344-213,321,498 | Alpha/beta-hydrolases superfamily protein |
| *Zm00001d007975* | 2 | 244,366,912-244,371,271 | Btb/poz domain-containing protein |
| *Zm00001d007962* | 2 | 244,254,163-244,256,107 | Myb-like dna-binding domain%2c shaqkyf class family protein |
| *Zm00001d007960* | 2 | 244,109,455-244,114,715 | T-complex protein 1 subunit epsilon |
| *Zm00001d007959* | 2 | 244,092,998-244,094,473 | Unknown |
| *Zm00001d007967* | 2 | 244,318,070-244,318,676 | Unknown |
| *Zm00001d007973* | 2 | 244,357,783-244,359,645 | Galactoside 2-alpha-l-fucosyltransferase |
| *Zm00001d007840* | 2 | 240,624,534-240,627,667 | Ap2-like ethylene-responsive transcription factor ant |
| *Zm00001d007978* | 2 | 244,407,619-244,413,284 | Chromatin complex subunit a101 |
| *Zm00001d007971* | 2 | 244,349,184-244,350,764 | Osjnbb0004g23.8 protein; osjnbb0085f13.3 protein |
| *Zm00001d007961* | 2 | 244,247,294-244,251,653 | Unknown |
| *Zm00001d007963* | 2 | 244,274,382-244,275,960 | Unknown |
| *Zm00001d007977* | 2 | 244,405,957-244,406,451 | Unknown |
| *Zm00001d007966* | 2 | 244,311,542-244,316,936 | Sudh4;succinate dehydrogenase4: |
| *Zm00001d007839* | 2 | 240,606,690-240,609,986 | Rna-binding protein 1 |
| *Zm00001d007974* | 2 | 244,365,350-244,365,931 | Unknown |
| *Zm00001d007972* | 2 | 244,351,539-244,356,560 | Tetratricopeptide repeat (tpr)-like superfamily protein |
| *Zm00001d007969* | 2 | 244,325,757-244,326,830 | Cold-regulated 413 plasma membrane protein 2 |
| *Zm00001d042263* | 3 | 157,976,446-157,978,862 | Inducer of cbf expression 2 |
| *Zm00001d042264* | 3 | 158,018,621-158,023,262 | Aspartokinase 1 chloroplastic |
| *Zm00001d042260* | 3 | 157,879,476-157,881,240 | Chloroplastic quinone-oxidoreductase |
| *Zm00001d042265* | 3 | 158,026,796-158,027,681 | Unknown |
| *Zm00001d042262* | 3 | 157,883,650-157,895,430 | Retrovirus-related pol polyprotein line-1 |
| *Zm00001d042267* | 3 | 158,098,798-158,103,660 | Auxin response factor 10 |
| *Zm00001d042266* | 3 | 158,052,163-158,056,995 | Eukaryotic initiation factor 3 gamma subunit family protein |
| *Zm00001d043418* | 3 | 199,094,453-199,105,329 | La-related protein 6a |
| *Zm00001d043414* | 3 | 199,050,966-199,054,946 | Unknown |
| *Zm00001d043404* | 3 | 198,914,206-198,921,192 | Dna polymerase eta |
| *Zm00001d043422* | 3 | 199,163,496-199,164,221 | Ring-h2 finger protein atl2k |
| *Zm00001d043411* | 3 | 199,019,233-199,021,284 | Ga2ox3;gibberellin 2-oxidase3: |
| *Zm00001d043425* | 3 | 199,386,458-199,395,287 | Unknown |
| *Zm00001d043419* | 3 | 199,107,176-199,108,703 | Unknown |
| *Zm00001d043416* | 3 | 199,087,596-199,089,376 | Unknown |
| *Zm00001d043420* | 3 | 199,110,559-199,113,757 | Basic leucine zipper 34 |
| *Zm00001d043407* | 3 | 198,975,889-198,981,155 | Sgs domain-containing protein |
| *Zm00001d043410* | 3 | 199,003,871-199,008,830 | P-loop containing nucleoside triphosphate hydrolases superfamily protein |
| *Zm00001d043405* | 3 | 198,921,272-198,923,752 | Pentatricopeptide repeat-containing protein |
| *Zm00001d043421* | 3 | 199,156,004-199,157,305 | Unknown |
| *Zm00001d043415* | 3 | 199,055,900-199,056,426 | Uncharacterised conserved protein |
| *Zm00001d043412* | 3 | 199,026,006-199,039,277 | Unknown |
| *Zm00001d043406* | 3 | 198,944,183-198,946,076 | Interactor of constitutive active rops 1 |
| *Zm00001d043403* | 3 | 198,911,644-198,912,362 | Heavy metal transport/detoxification superfamily protein |
| *Zm00001d043413* | 3 | 199,047,486-199,047,710 | Unknown |
| *Zm00001d050021* | 4 | 60,281,640-60,287,213 | Abh3;abscisic acid 8'-hydroxylase3 |
| *Zm00001d014485* | 5 | 49,842,078-49,843,394 | Zinc finger protein-related |
| *Zm00001d014487* | 5 | 49,948,374-49,954,685 | Gdsl esterase/lipase |
| *Zm00001d014486* | 5 | 49,915,116-49,917,898 | Phosphosulfolactate synthase-related protein |
| *Zm00001d014490* | 5 | 50,037,295-50,041,318 | Unknown |
| *Zm00001d014482* | 5 | 49,749,761-49,762,625 | Replication protein-like |
| *Zm00001d014492* | 5 | 50,147,875-50,148,531 | Unknown |
| *Zm00001d014489* | 5 | 50,002,094-50,007,362 | Putative beta-glucosidase 41 |
| *Zm00001d014491* | 5 | 50,064,948-50,065,346 | Ef-hand ca2+-binding protein ccd1 |
| *Zm00001d014488* | 5 | 49,999,383-50,001,892 | 50s ribosomal protein l24 chloroplastic |
| *Zm00001d014630* | 5 | 56,629,737-56,631,089 | Homeobox-leucine zipper protein hat9 |
| *Zm00001d014629* | 5 | 56,627,074-56,628,308 | Homeobox-leucine zipper protein hat9 |
| *Zm00001d014628* | 5 | 56,552,093-56,553,397 | Homeobox-leucine zipper protein hat9 |
| *Zm00001d014632* | 5 | 56,752,087-56,756,057 | Superoxide dismutase |
| *Zm00001d014626* | 5 | 56,455,238-56,460,704 | Ribosomal rna processing brix domain protein |
| *Zm00001d019117* | 7 | 17,793,574-17,795,478 | Unknown |
| *Zm00001d019123* | 7 | 17,937,714-17,949,491 | Survival protein sure-like phosphatase/nucleotidase |
| *Zm00001d019116* | 7 | 17,758,679-17,759,647 | Ethylene-responsive transcription factor rap2-11 |
| *Zm00001d019122* | 7 | 17,900,843-17,901,808 | Unknown |
| *Zm00001d020720* | 7 | 130,092,092-130,094,267 | Unknown |
| *Zm00001d020721* | 7 | 130,105,577-130,107,324 | Galactoside 2-alpha-l-fucosyltransferase |
| *Zm00001d020713* | 7 | 129,773,310-129,774,515 | Unknown |
| *Zm00001d021098* | 7 | 142,745,483-142,746,040 | Unknown |
| *Zm00001d020717* | 7 | 129,916,943-129,919,846 | Abh4;abscisic acid 8'-hydroxylase4: |
| *Zm00001d020719* | 7 | 130,049,746-130,054,538 | Protein weak chloroplast movement under blue light 1 |
| *Zm00001d020714* | 7 | 129,797,898-129,801,599 | Heat shock factor protein 4 |
| *Zm00001d020718* | 7 | 130,047,693-130,048,670 | Unknown |
| *Zm00001d010455* | 8 | 115,967,122-115,979,410 | Unknown |
| *Zm00001d010458* | 8 | 116,141,888-116,147,596 | Protein kinase superfamily protein |
| *Zm00001d010459* | 8 | 116,264,102-116,265,454 | Putative cbl-interacting protein kinase family protein |
| *Zm00001d010454* | 8 | 115,914,488-115,918,396 | Mannosyl-oligosaccharide 12-alpha-mannosidase mns3 |
| *Zm00001d010456* | 8 | 116,048,099-116,074,297 | Unknown |
| *Zm00001d011365* | 8 | 148,696,781-148,699,784 | Alpha/beta-hydrolases superfamily protein |
| *Zm00001d011363* | 8 | 148,506,457-148,509,460 | Putative oxidoreductase%2c aldo/keto reductase family protein |
| *Zm00001d011360* | 8 | 148,421,209-148,436,906 | Cenpcb protein |
| *Zm00001d011362* | 8 | 148,481,033-148,484,238 | Psbp domain-containing protein 5 chloroplastic |
| *Zm00001d011366* | 8 | 148,702,797-148,711,327 | Atp-dependent zinc metalloprotease ftsh 11 chloroplastic/mitochondrial |
| *Zm00001d011369* | 8 | 148,782,535-148,785,419 | Pectin lyase-like superfamily protein |
| *Zm00001d011364* | 8 | 148,605,323-148,613,271 | Probable protein phosphatase 2c 76 |
| *Zm00001d011370* | 8 | 148,833,239-148,839,951 | Hydrolase%2c hydrolyzing o-glycosyl compound |
| *Zm00001d011368* | 8 | 148,751,586-148,753,559 | Formate dehydrogenase chloroplastic/mitochondrial |
| *Zm00001d012710* | 8 | 179,073,312-179,076,818 | Sgs domain-containing protein |
| *Zm00001d012729* | 8 | 179,415,114-179,417,574 | Protein nuclear fusion defective 6 chloroplastic/mitochondrial |
| *Zm00001d012708* | 8 | 179,043,085-179,046,513 | Pentatricopeptide repeat-containing protein |
| *Zm00001d012728* | 8 | 179,391,080-179,396,140 | Putative duf1421 domain family protein |
| *Zm00001d012725* | 8 | 179,214,402-179,228,713 | Transcription factor tcp4 |
| *Zm00001d012720* | 8 | 179,173,501-179,174,214 | E3 ubiquitin-protein ligase atl41 |
| *Zm00001d012717* | 8 | 179,106,798-179,113,012 | Potassium channel kat1 |
| *Zm00001d012714* | 8 | 179,100,848-179,101,078 | Unknown |
| *Zm00001d012709* | 8 | 179,050,887-179,052,423 | Interactor of constitutive active rops 1 |
| *Zm00001d012721* | 8 | 179,175,858-179,177,641 | Gibberellin receptor gid1 |
| *Zm00001d012730* | 8 | 179,422,235-179,422,728 | Unknown |
| *Zm00001d012711* | 8 | 179,078,879-179,081,493 | Dihydropyrimidinase |
| *Zm00001d012726* | 8 | 179,301,390-179,301,632 | Unknown |
| *Zm00001d012707* | 8 | 179,032,130-179,037,162 | Bifunctional riboflavin kinase/fmn phosphatase |
| *Zm00001d012712* | 8 | 179,086,672-179,088,998 | Ga2ox10;gibberellin 2-oxidase10: |
| *Zm00001d012719* | 8 | 179,164,455-179,168,169 | Bzip-type transcription factor |
| *Zm00001d012718* | 8 | 179,120,409-179,124,173 | Unknown |
| *Zm00001d010442* | 8 | 115,326,023-115,335,682 | Putative wall-associated receptor protein kinase family protein |
| *Zm00001d010446* | 8 | 115,490,413-115,493,023 | Endoglucanase 6 |
| *Zm00001d010445* | 8 | 115,433,371-115,435,609 | Abscisic acid receptor pyl9 |
| *Zm00001d010447* | 8 | 115,541,975-115,546,228 | Leucine-rich repeat (lrr) family protein |
| *Zm00001d010443* | 8 | 115,380,456-115,387,332 | Protein trichome birefringence-like 26 |
| *Zm00001d010450* | 8 | 115,694,102-115,697,499 | Gdsl esterase/lipase |
| *Zm00001d010448* | 8 | 115,603,884-115,610,448 | Putative leucine-rich repeat receptor-like protein kinase family protein%3b receptor protein kinase-like |
| *Zm00001d026291* | 10 | 142,982,967-142,990,366 | Met2;dna methyl transferase2 |
| *Zm00001d026288* | 10 | 142,887,361-142,891,016 | Mbd123;methyl binding domain123 |
| *Zm00001d026285* | 10 | 142,830,921-142,833,263 | Osjnba0070c17.17 protein |
| *Zm00001d026287* | 10 | 142,866,476-142,870,798 | Udp-n-acetylglucosamine diphosphorylase 2 |
| *Zm00001d026302* | 10 | 143,209,576-143,212,715 | Osjnba0008m17.14 protein |
| *Zm00001d036543* | 10 | 91,521,156-91,525,360 | Probable beta-14-xylosyltransferase irx14 |
| *Zm00001d026299* | 10 | 143,189,604-143,190,569 | Unknown |
| *Zm00001d026297* | 10 | 143,164,305-143,165,139 | Proline-rich family protein |
| *Zm00001d026300* | 10 | 143,197,942-143,203,163 | Ago2b;argonaute2b |
| *Zm00001d026296* | 10 | 143,156,312-143,161,817 | Trypsin family protein |
| *Zm00001d026289* | 10 | 142,956,463-142,961,794 | Potassium transporter 10 |
| *Zm00001d026295* | 10 | 143,128,213-143,138,549 | Unknown |
| *Zm00001d026293* | 10 | 143,077,541-143,089,471 | Gamma-aminobutyrate transaminase pop2 mitochondrial |
| *Zm00001d026298* | 10 | 143,168,656-143,170,119 | Putative heavy metal transport/detoxification superfamily protein |
| *Zm00001d026284* | 10 | 142,767,423-142,768,557 | Vacuolar atpase assembly integral membrane protein vma21-like domain |
| *Zm00001d026290* | 10 | 142,970,716-142,981,629 | Nad(p)-binding rossmann-fold superfamily protein |
| *Zm00001d026286* | 10 | 142,863,674-142,866,331 | Rps11;ribosomal protein s11 |

Chr., chromosome
