# Supplementary material for: Genome-wide association study uncovers new genetic loci and candidate genes underlying seed chilling-germination in maize
Source: PeerJ. 2021 Jun 28;9:e11707. doi: 10.7717/peerj.11707 (PMC8247712; doi:10.7717/peerj.11707)
Supplement: Supplemental Information 5 [file peerj-09-11707-s005.docx]

**Supplementary Table S5.** Results of candidate gene association analysis in this study.

| Candidate gene | SNP | Chr. | Allele type | | | Position (bp) | P.value |
| --- | --- | --- | --- | --- | --- | --- | --- |
| *Zm00001d050021* | SNP-4-60279645 | 4 | | G/T | 60,279,645 | | 4.78E-01 |
|  | SNP-4-60281430 | 4 | | C/T | 60,281,430 | | 1.22E-01 |
|  | SNP-4-60282052 | 4 | | C/T | 60,282,052 | | 1.22E-02 |
|  | SNP-4-60282131 | 4 | | G/C | 60,282,131 | | 8.24E-01 |
|  | SNP-4-60282162 | 4 | | T/G | 60,282,162 | | 8.00E-01 |
|  | SNP-4-60282276 | 4 | | G/T | 60,282,276 | | 6.85E-02 |
|  | SNP-4-60282315 | 4 | | C/T | 60,282,315 | | 8.99E-02 |
|  | SNP-4-60282408 | 4 | | G/A | 60,282,408 | | 1.15E-01 |
|  | SNP-4-60283214 | 4 | | G/C | 60,283,214 | | 2.97E-01 |
|  | SNP-4-60283489 | 4 | | C/G | 60,283,489 | | 3.26E-01 |
|  | SNP-4-60283545 | 4 | | T/C | 60,283,545 | | 7.56E-02 |
|  | SNP-4-60288956 | 4 | | C/G | 60,288,956 | | 4.84E-02 |
| *Zm00001d019123* | SNP-7-17937736 | 7 | | T/G | 17,937,736 | | 2.55E-02 |
|  | SNP-7-17938301 | 7 | | C/T | 17,938,301 | | 6.24E-02 |
|  | SNP-7-17939242 | 7 | | C/T | 17,939,242 | | 6.24E-02 |
|  | SNP-7-17939285 | 7 | | G/T | 17,939,285 | | 5.73E-02 |
| *Zm00001d019116* | SNP-7-17757399 | 7 | | G/C | 17,757,399 | | 6.29E-01 |
|  | SNP-7-17757535 | 7 | | A/G | 17,757,535 | | 3.75E-01 |
|  | SNP-7-17757649 | 7 | | T/C | 17,757,649 | | 1.85E-01 |
|  | SNP-7-17757709 | 7 | | A/T | 17,757,709 | | 1.73E-01 |
|  | SNP-7-17758874 | 7 | | A/G | 17,758,874 | | 6.24E-02 |
|  | SNP-7-17759020 | 7 | | C/T | 17,759,020 | | 6.24E-02 |
|  | IND-7-17759166 | 7 | | -/G | 17,759,166 | | 5.73E-02 |
|  | SNP-7-17759531 | 7 | | G/A | 17,759,531 | | 7.73E-02 |
|  | SNP-7-17759540 | 7 | | G/T | 17,759,540 | | 1.62E-01 |
|  | SNP-7-17759593 | 7 | | C/G | 17,759,593 | | 4.68E-01 |
|  | SNP-7-17759636 | 7 | | A/C | 17,759,636 | | 2.49E-01 |
| *Zm00001d010454* | SNP-8-115913550 | 8 | | C/A | 115,913,550 | | 2.43E-03 |
|  | SNP-8-115914234 | 8 | | A/G | 115,914,234 | | 7.04E-02 |
|  | SNP-8-115914606 | 8 | | C/T | 115,914,606 | | 1.95E-03 |
|  | SNP-8-115915557 | 8 | | A/T | 115,915,557 | | 9.63E-02 |
|  | SNP-8-115916330 | 8 | | G/T | 115,916,330 | | 6.17E-02 |
|  | SNP-8-115916675 | 8 | | C/T | 115,916,675 | | 1.97E-01 |
|  | SNP-8-115917258 | 8 | | C/A | 115,917,258 | | 7.29E-02 |
| *Zm00001d010458* | SNP-8-116142647 | 8 | | G/A | 116,142,647 | | 1.70E-01 |
|  | SNP-8-116142679 | 8 | | T/C | 116,142,679 | | 3.04E-01 |
|  | SNP-8-116142689 | 8 | | T/G | 116,142,689 | | 3.04E-01 |
|  | SNP-8-116143077 | 8 | | G/A | 116,143,077 | | 1.79E-01 |
|  | SNP-8-116143136 | 8 | | T/C | 116,143,136 | | 2.29E-01 |
|  | SNP-8-116144738 | 8 | | G/A | 116,144,738 | | 6.06E-02 |
|  | SNP-8-116144948 | 8 | | T/C | 116,144,948 | | 2.70E-01 |
|  | SNP-8-116145007 | 8 | | G/A | 116,145,007 | | 1.22E-01 |
|  | SNP-8-116145034 | 8 | | G/A | 116,145,034 | | 1.22E-01 |
|  | SNP-8-116145286 | 8 | | A/C | 116,145,286 | | 2.05E-01 |
|  | SNP-8-116145327 | 8 | | G/A | 116,145,327 | | 2.07E-01 |
|  | SNP-8-116145835 | 8 | | C/T | 116,145,835 | | 2.08E-01 |
|  | SNP-8-116146382 | 8 | | G/T | 116,146,382 | | 2.44E-01 |
|  | SNP-8-116146414 | 8 | | G/C | 116,146,414 | | 3.54E-01 |
|  | SNP-8-116146687 | 8 | | G/A | 116,146,687 | | 1.32E-01 |
|  | SNP-8-116147619 | 8 | | A/G | 116,147,619 | | 3.09E-03 |
|  | SNP-8-116147732 | 8 | | A/G | 116,147,732 | | 3.88E-01 |
|  | SNP-8-116148568 | 8 | | C/G | 116,148,568 | | 8.63E-03 |
|  | SNP-8-116149100 | 8 | | T/C | 116,149,100 | | 1.97E-01 |
|  | SNP-8-116149207 | 8 | | G/A | 116,149,207 | | 8.38E-02 |
|  | SNP-8-116149426 | 8 | | A/G | 116,149,426 | | 7.27E-02 |
| *Zm00001d010459* | SNP-8-116261524 | 8 | | T/C | 116,261,524 | | 1.85E-01 |
|  | SNP-8-116262506 | 8 | | G/A | 116,262,506 | | 8.00E-02 |
|  | SNP-8-116262789 | 8 | | C/A | 116,262,789 | | 2.90E-03 |
|  | SNP-8-116264478 | 8 | | A/G | 116,264,478 | | 2.25E-01 |
|  | SNP-8-116264543 | 8 | | C/T | 116,264,543 | | 2.36E-01 |
|  | SNP-8-116264686 | 8 | | T/C | 116,264,686 | | 7.61E-02 |
|  | IND-8-116266748 | 8 | | G/- | 116,266,748 | | 7.05E-01 |
|  | SNP-8-116266856 | 8 | | A/T | 116,266,856 | | 6.82E-02 |
|  | SNP-8-116266861 | 8 | | G/A | 116,266,861 | | 7.82E-02 |
|  | SNP-8-116267372 | 8 | | C/T | 116,267,372 | | 1.32E-01 |
|  | SNP-8-116267516 | 8 | | C/T | 116,267,516 | | 1.96E-01 |
|  | IND-8-116267635 | 8 | | G/- | 116,267,635 | | 1.65E-01 |
|  | SNP-8-116268236 | 8 | | T/C | 116,268,236 | | 1.73E-01 |
|  | SNP-8-116268357 | 8 | | C/G | 116,268,357 | | 1.33E-01 |
|  | SNP-8-116268359 | 8 | | A/G | 116,268,359 | | 1.33E-01 |

Chr., chromosome
