# Supplementary material for: Genome-wide association study uncovers new genetic loci and candidate genes underlying seed chilling-germination in maize
Source: PeerJ. 2021 Jun 28;9:e11707. doi: 10.7717/peerj.11707 (PMC8247712; doi:10.7717/peerj.11707)
Supplement: Supplemental Information 6 [file peerj-09-11707-s006.docx]

**Supplementary Table S6.** Our identified significant SNPs overlapping with QTLs/SNPs under cold-germination identified in previous studies.

| Our study | |  | Previous study | |  |
| --- | --- | --- | --- | --- | --- |
| Trait | SNP | Chr. | QTLs/SNPs (cM) | Associated taits | Reference |
| RRS | PZE-102118136 | 2 | 98-158 | Straw dry weight | Leipner et al. |
| SL | PZE-102193367 | 2 | 216-260 | Straw dry weight | Leipner et al. |
| FG | PZE-105052784 | 5 | 43-88 | Female flowering | Leipner et al. |
| TG | PZE-105056721 | 5 | 43-88 | Female flowering | Leipner et al. |
| FG, TG | PZE-107018981 | 7 | 0-52 | Female flowering | Leipner et al. |
| FG, TG | PZE-107018981 | 7 | 6-58 | Shoot dry weight | Leipner et al. |
| FG, TG | PZE-107018981 | 7 | 0-58 | Straw dry weight | Leipner et al. |
| FG, TG | PZE-107018981 | 7 | 4-62 | Ear dry weight | Leipner et al. |
| FG, TG | PZE-107018981 | 7 | 0-20 | Seminal Weight | Hund et al. |
| FG, TG | PZE-107018981 | 7 | 0-20 | PrAx Length | Hund et al. |
| RL, SL | PZE-104042136 | 4 | 0-69 | SeLat Length | Hund et al. |
| FG | PZE-103096466 | 3 | 100-160 | PrAx Diameter | Hund et al. |
| RRS | PZE-108086767 | 1 | 218,962,026 | Fresh root weight | Huang et al. |
| RL | PZE-107112738 | 7 | 161,974,361 | Fresh root weight | Huang et al. |

Chr., chromosome
