# Supplementary figures and images for: Genome-wide association study uncovers new genetic loci and candidate genes underlying seed chilling-germination in maize
Source: PeerJ. 2021 Jun 28;9:e11707. doi: 10.7717/peerj.11707 (PMC8247712; doi:10.7717/peerj.11707)

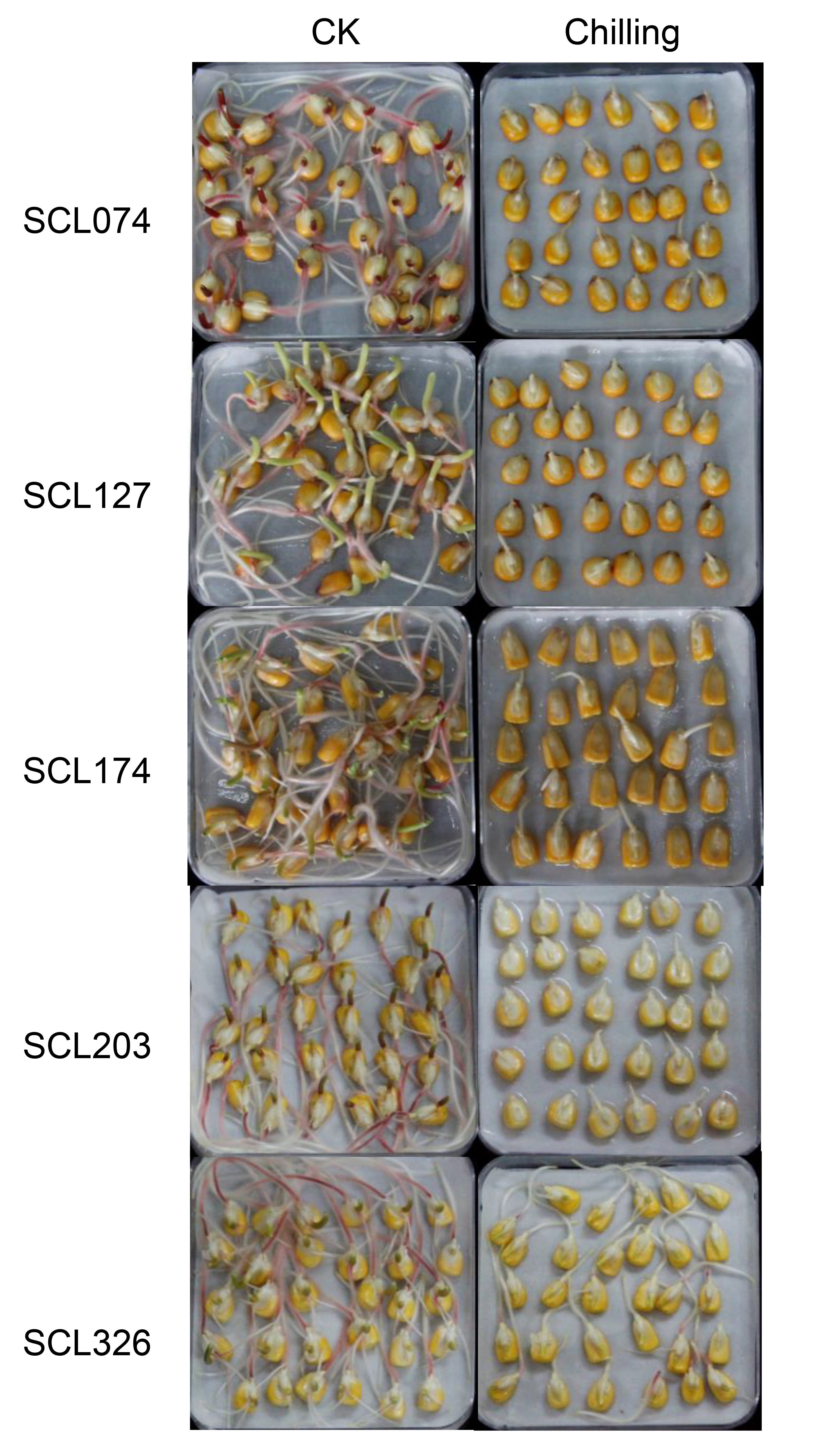

Supplement: Supplemental Information 7 [file peerj-09-11707-s007.png]

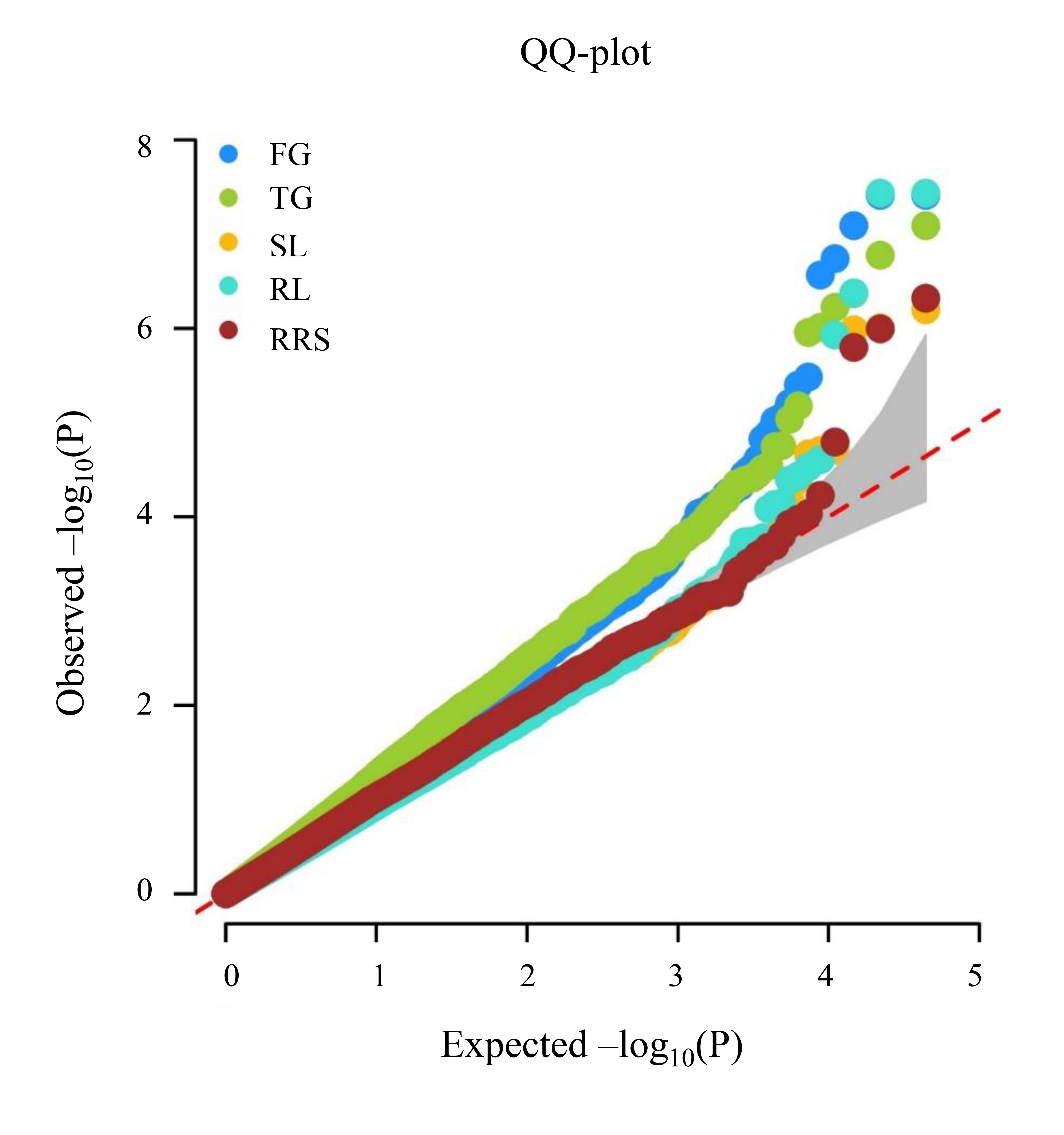

Supplement: Supplemental Information 8 [file peerj-09-11707-s008.png]

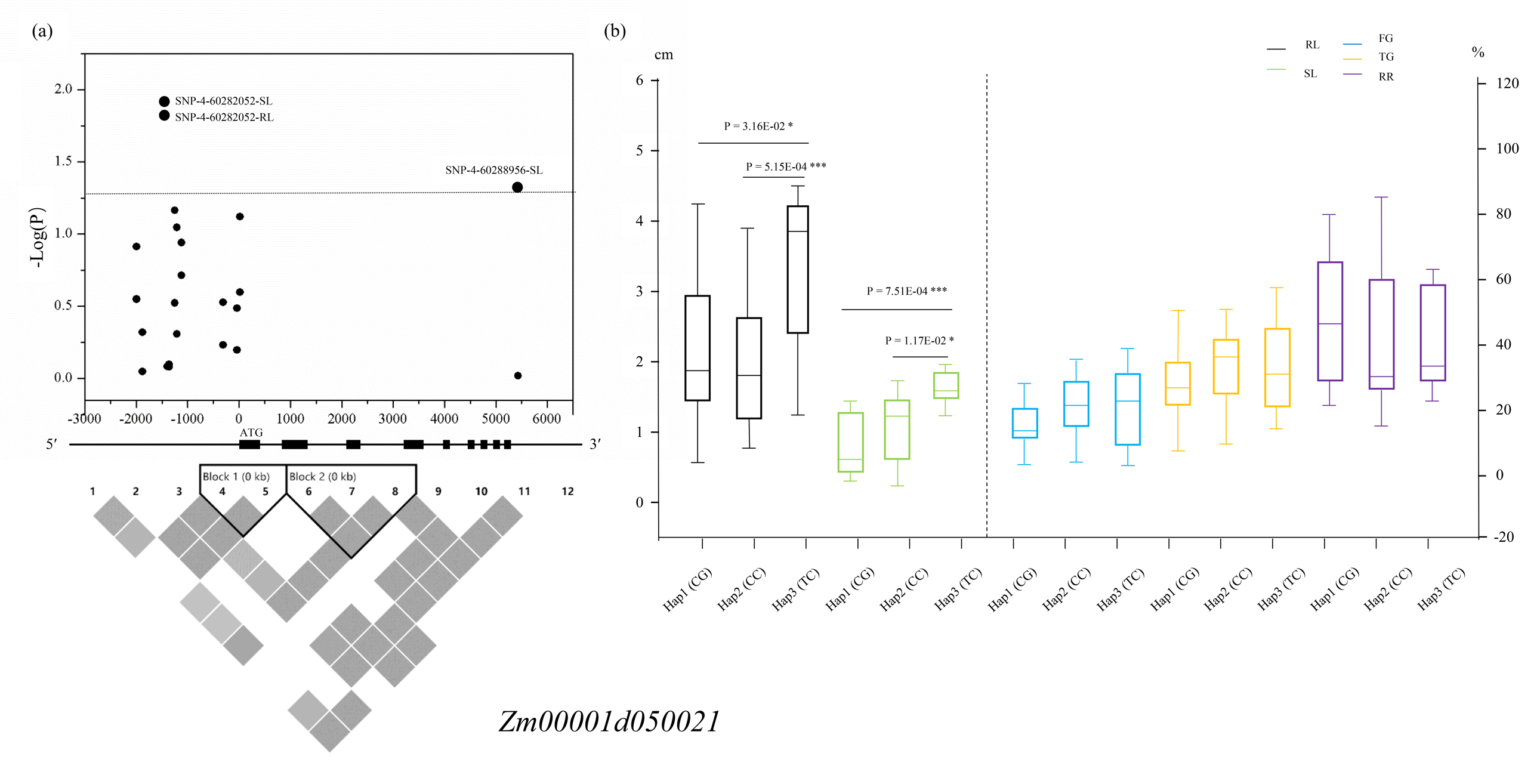

Supplement: Supplemental Information 9 — (A) Dark dots above the dotted line display the significantly ( P < 0.05) associated SNPs. The structure of the gene Zm00001d050021 is displayed in the middle. The extrons are represented by filled dark boxes, the promoter and 5′-UTR are shown by the left dark line, the introns were denoted by the dark lines between the filled dark boxes, and the 3′-UTR are displayed by the right dark line. The bottom image shows the pairwise LDs between the markers. (B) Comparison of chilling-germination phenotypic performance between three haplotypes. * and *** represent the significant levels of P < 0.05 and P < 0.001, respectively. Hap, haplotypes; FG, Germination rate at 5 d; TG, Germination rate at 10 d; RL, Root length at 10 d; SL, Shoot length at 10 d; RRS, Ratio of root length to shoot length. [file peerj-09-11707-s009.png]

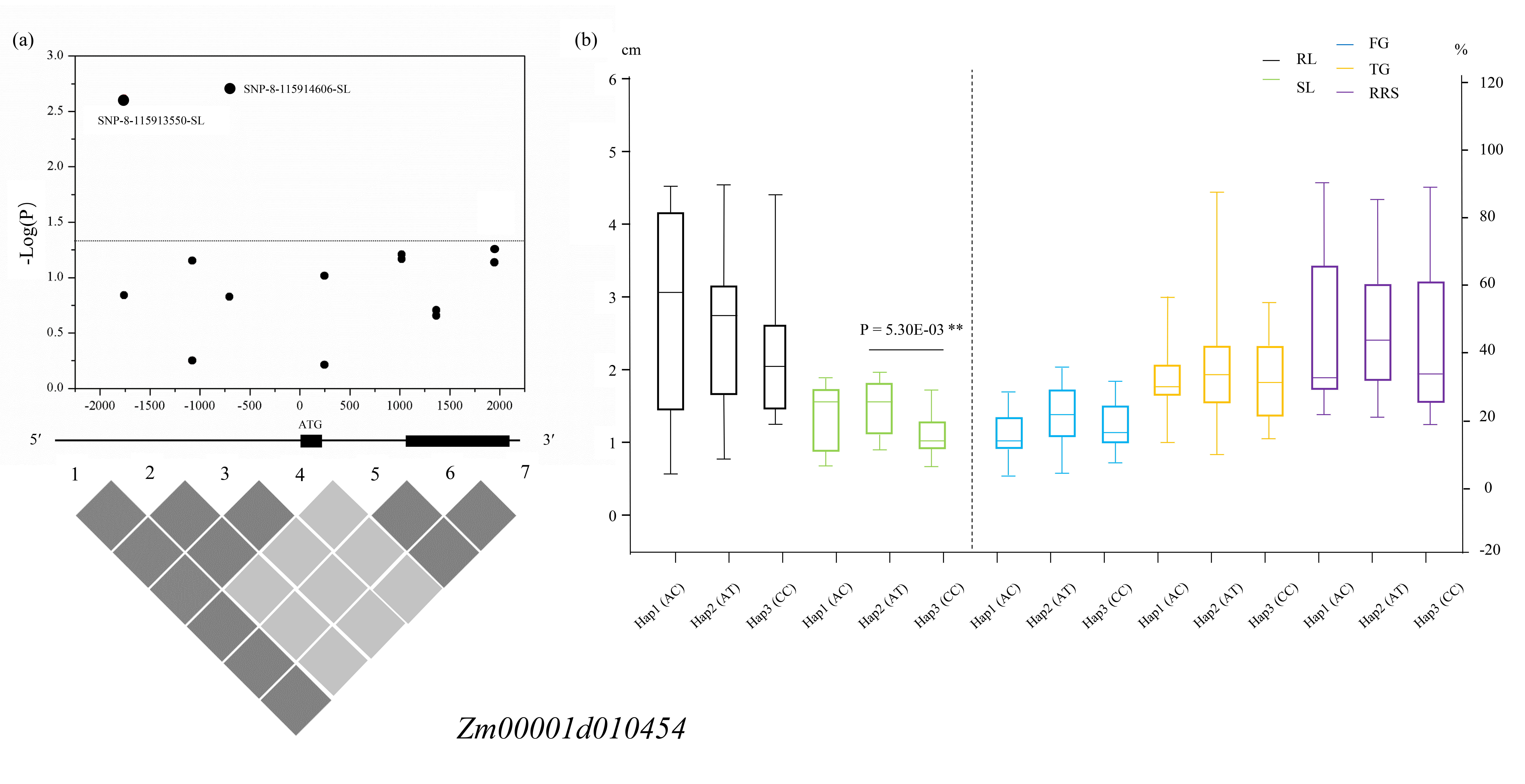

Supplement: Supplemental Information 10 — (A) Dark dots above the dotted line display the significantly ( P < 0.05) associated SNPs. The structure of the gene Zm00001d010454 is displayed in the middle. The extrons are represented by filled dark boxes, the promoter and 5′-UTR are shown by the left dark line, the introns were denoted by the dark lines between the filled dark boxes, and the 3′-UTR are displayed by the right dark line. The bottom image shows the pairwise LDs between the markers. (B) Comparison of chilling-germination phenotypic performance between three haplotypes. ** represent the significant level of P < 0.05. Hap, haplotypes; FG, Germination rate at 5 d; TG, Germination rate at 10 d; RL, Root length at 10 d; SL, Shoot length at 10 d; RRS, Ratio of root length to shoot length. [file peerj-09-11707-s010.png]

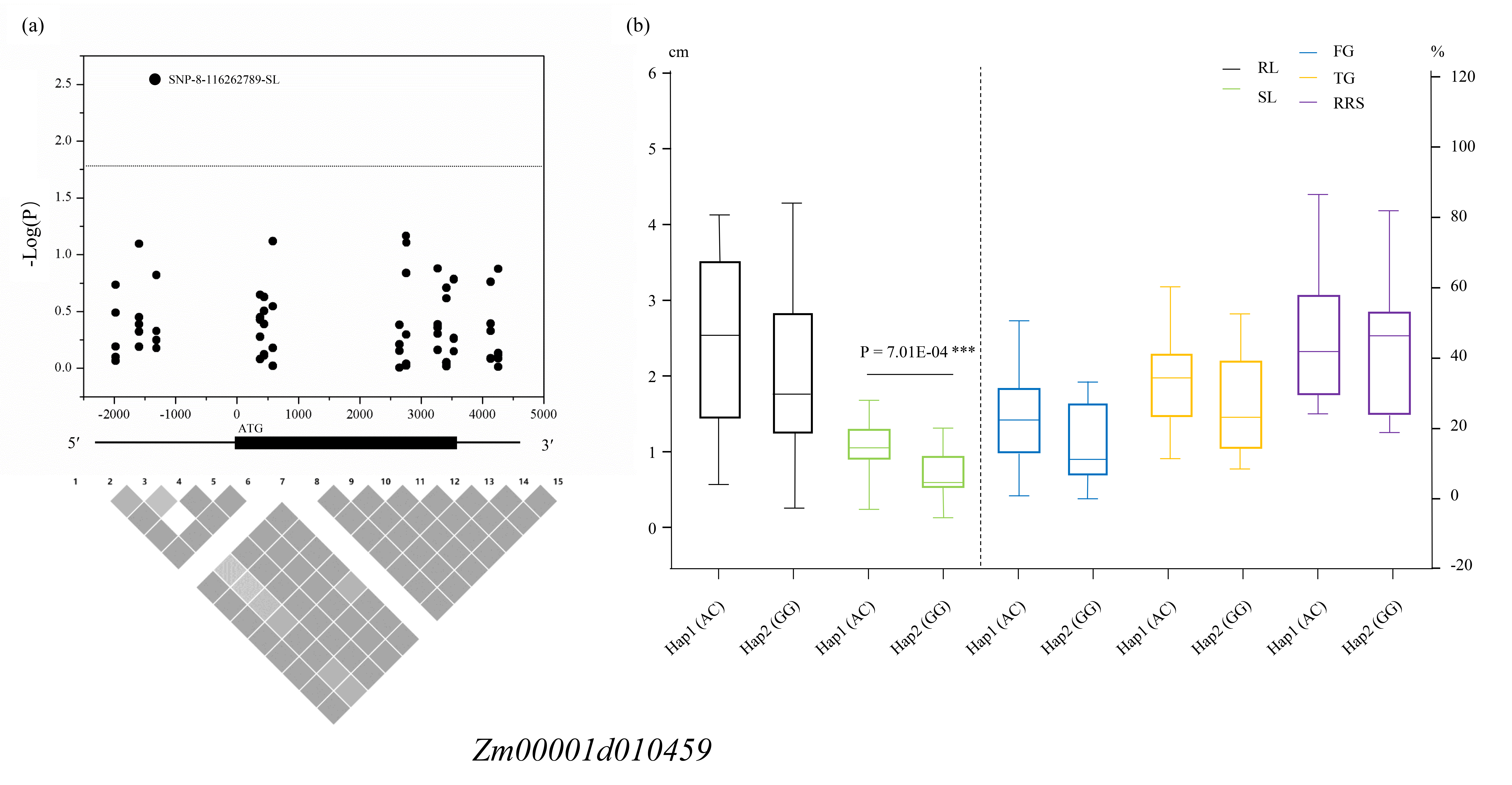

Supplement: Supplemental Information 11 — (A) Dark dot above the dotted line shows significant ( P < 0.05) SNP. The structure of the gene Zm00001d010459 is displayed in the middle. The extrons are represented by filled dark boxes, the promoter and 5′-UTR are shown by the left dark line, the introns were denoted by the dark lines between the filled dark boxes, and the 3′-UTR are displayed by the right dark line. The bottom image shows the pairwise LDs between the markers. (B) Comparison of chilling-germination phenotypic performance between two haplotypes. *** represent the significant level of P < 0.001. Hap, haplotypes; FG, Germination rate at 5 d; TG, Germination rate at 10 d; RL, Root length at 10 d; SL, Shoot length at 10 d; RRS, Ratio of root length to shoot length. [file peerj-09-11707-s011.png]
